# Assessment of phylogenetic approaches to study the timing of recombination cessation on sex chromosomes

**DOI:** 10.1111/jeb.14068

**Published:** 2022-07-27

**Authors:** Hongkai Zhang, Hanna Sigeman, Bengt Hansson

**Affiliations:** ^1^ Department of Biology Lund University Lund Sweden

**Keywords:** evolutionary strata, phylogenetic approaches, sex chromosome, timing of recombination cessation

## Abstract

The evolution of sex chromosomes is hypothesized to be punctuated by consecutive recombination cessation events, forming “evolutionary strata” that ceased to recombine at different time points. The demarcation of evolutionary strata is often assessed by estimates of the timing of recombination cessation (*t*_RC_) along the sex chromosomes, commonly inferred from the level of synonymous divergence or with species phylogenies at gametologous (X‐Y or Z‐W) sequence data. However, drift and selection affect sequences unpredictably and introduce uncertainty when inferring *t*_RC_. Here, we assess two alternative phylogenetic approaches to estimate *t*_RC_; (i) the expected likelihood weight (ELW) approach that finds the most likely topology among a set of hypothetical topologies and (ii) the BEAST approach that estimates *t*_RC_ with specified calibration priors on a reference species topology. By using Z and W gametologs of an old and a young evolutionary stratum on the neo‐sex chromosome of Sylvioidea songbirds, we show that the ELW and BEAST approaches yield similar *t*_RC_ estimates, and that both outperform two frequently applied approaches utilizing synonymous substitution rates (dS) and maximum likelihood (ML) trees, respectively. Moreover, we demonstrate that both ELW and BEAST provide more precise *t*_RC_ estimates when sequences of multiple species are included in the analyses.

## Graphical abstract

We assess alternative approaches to estimate the timing of recombination cessation (*t*_RC_) along sex chromosomes using sequence data of Z and W gametologs located in old (ancestral) and young (added) regions of the neo‐sex chromosome of Sylvioidea songbirds. We show that the expected likelihood weight (ELW) and BEAST approaches yield similar *t*_RC_ estimates, and that both outperform two frequently applied approaches utilising synonymous substitution rates (dS) and maximum likelihood (ML) trees, respectively.

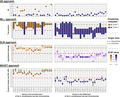

## 1 INTRODUCTION

Research over the last century has revealed a diversity of sex chromosomes in both plants and animals, ranging from homomorphic male‐heterogametic XY system in some fishes to heteromorphic female‐heterogametic ZW system in birds (Abbott et al., 2017; Bachtrog et al., 2011). The canonical model of sex chromosome evolution suggests that sex chromosomes evolved from autosomes after acquiring one or several sex‐determining genes, and that recombination cessation was selectively favored on the proto‐sex chromosomes to facilitate cosegregation between the sex‐determining allele(s) and sexually antagonistic variation at closely linked loci (Bachtrog et al., 2011; Charlesworth et al., 2005). The established non‐recombining region around the sex‐determining genes can then expand into larger sex‐specific regions until, as observed in the most mammals and birds, almost the entire chromosome lacks recombination (Cortez et al., 2014; Zhou et al., 2014). This expansion has been hypothesized to occur stepwise through relatively extensive recombination cessation events at different time points, forming the so‐called “evolutionary strata” that can be shown by spatial clusters of X‐Y or Z‐W gametologs along the sex chromosomes with similar divergence estimates (Bellott et al., 2014; Lahn & Page, 1999; Wright et al., 2016).

Evolutionary strata were firstly reported in humans by a divergence analysis of 19 X‐Y gametologs suggesting four strata, implicating four recombination cessation events (Lahn & Page, 1999). More recently, evolutionary strata were also reported in other eutherians and in monotremes (Bellott et al., 2014; Cortez et al., 2014), as well as in birds (Zhou et al., 2014; Wang et al., 2021). In mammals, two strata were reported in marsupials and rodents, three in bovids, and four in primates (Bellott et al., 2014; Lahn & Page, 1999). Also on the avian Z chromosomes, up to four evolutionary strata (S0‐S3) have been reported. As expected, the putative sex‐determining gene in birds, *DMRT1* (Smith et al., 2009), is located in the oldest stratum, S0 (Zhou et al., 2014). However, the number and order of evolutionary strata on the Z chromosome differ between bird lineages, from the S0‐S1/S2 in Paleognaths to the mosaic ordering of S0‐S3 in Neognaths (Nam & Ellegren, 2008; Xu et al., 2019; Zhou et al., 2014), due to lineage‐specific chromosomal rearrangements such as large inversions during avian evolution (Zhou et al., 2014). The hypothesized stepwise expansion of recombination cessation along heteromorphic sex chromosomes has, however, been challenged by data of the added part of the neo‐sex chromosomes in sticklebacks and warblers, where a more local and mosaic process for the progression of recombination cessation was instead suggested (Natri et al., 2013; Sigeman et al., 2021).

The general principle to study evolutionary strata is to estimate the timing of recombination cessation (*t*_RC_) along the sex chromosomes. After recombination ceases, the gametologs (X‐Y or Z‐W) follow unique and different evolutionary trajectories. In particular, the sex‐limited, hemizygous Y chromosome in mammals and W chromosome in birds experience significantly altered selection regime and demography, which may eventually lead to substantial chromosomal degeneration where only genes under strong purifying selection will survive over long evolutionary time scales (Bachtrog, 2013; Bellott et al., 2014; Sigeman et al., 2021). As the gametologs will start to differentiate only after recombination has stopped, *t*_RC_ can be inferred by estimating the time since the gametologs diverged (Bergero & Charlesworth, 2009). A frequently applied method to estimate *t*_RC_ is to measure the rate of synonymous substitutions (dS) between gametologs (Lahn & Page, 1999; Nam & Ellegren, 2008; Peichel et al., 2020). The dS approach is based on the assumption of neutrality, that is, that synonymous substitutions occur at an approximately constant rate in the DNA sequences and can, therefore, be used to date evolutionary events (Nam & Ellegren, 2008). Thus, dS between gametologs should be proportional to the time since they got isolated from one another. However, it is debated whether all synonymous substitutions are actually neutral (Chamary et al., 2006; Shields et al., 1988; Williams & Hurst, 2002). For instance, dS can be affected by biased codon usage, GC content, biased gene conversion (Smith & Eyre‐Walker, 2001) and site‐specific mutation rates (Berg, 1999). Moreover, there is evidence that dS can be affected by saturation, that is, additional mutations on already mutated sites (Smith & Smith, 1996). Together, the possible non‐neutrality of some synonymous substitutions may undermine dS as a proxy of *t*_RC_.

A conceptually different approach to estimate *t*_RC_ is by applying a phylogenetic framework, which embodies the evolutionary relationship between gametologs of several species in a graph consisting of branches and nodes (Semple et al., 2003). This is based on the idea that early and late recombination suppressed genes along the sex chromosome will exhibit different topologies. For example, when the gametologs of a set of species cluster exclusively according to chromosome type rather than to species, recombination cessation predates even the oldest speciation event in the topology, which thereby provides a minimum age of the stratum where the gene is located. Maximum likelihood (ML) is a widely applied methodology that aims at finding the most likely phylogenetic tree by maximizing a likelihood function given the alignment and tree parameters. A noticeable constraint of the ML approach is that the precision of *t*_RC_ is determined by the number of dated speciation events in the topology; the fewer the species, the larger the time intervals between branches. Moreover, the topology of a gene tree may be difficult to resolve owing to gene duplication, saturation, incomplete lineage sorting (Doyle, 1992; Maddison, 1997; Sanderson & Shaffer, 2002), too few informative sites because of short alignments, etc. Unresolved topology of a gene tree makes interpreting *t*_RC_ difficult because it fails to conform to any of the topologies we expect for different recombination cessation scenarios. By statistical evaluation of the phylogenetic tree (e.g., by bootstrapping; Efron et al., 1996), and by collapsing unresolved branches, one can however obtain a confident topology with which a *t*_RC_ range of uniform probability distribution can then be inferred (Sigeman et al., 2021).

Here, we introduce two alternative phylogenetic approaches to estimate *t*_RC_ on sex chromosomes; the expected likelihood weight (ELW) approach and the BEAST approach. In the ELW approach, the ELW values measure the relative support of each topology in a finite set of hypothetical topologies (Strimmer & Rambaut, 2002). When it comes to sex chromosomes, topologies representing all possible recombination cessation scenarios are constructed by arranging gametologs of one or more focal species on a reference species topology, and the topology with the highest ELW value signifies the most probable *t*_RC_. The BEAST approach estimates *t*_RC_ by applying Markov chain Monte Carlo (MCMC) algorithm (Bouckaert et al., 2019) to model the posterior probability distribution for the divergence node between the gametologs of a focal species in a reference species topology with specified calibration priors on the other nodes. Compared with other phylogenetic approaches, the ELW and BEAST approaches are able to pinpoint the most probable *t*_RC_, and to transform the uncertainty of the topology of a gene tree to an uncertainty around *t*_RC_, thereby providing an unbiased interpretation of when recombination ceased.

**FIGURE 1:**
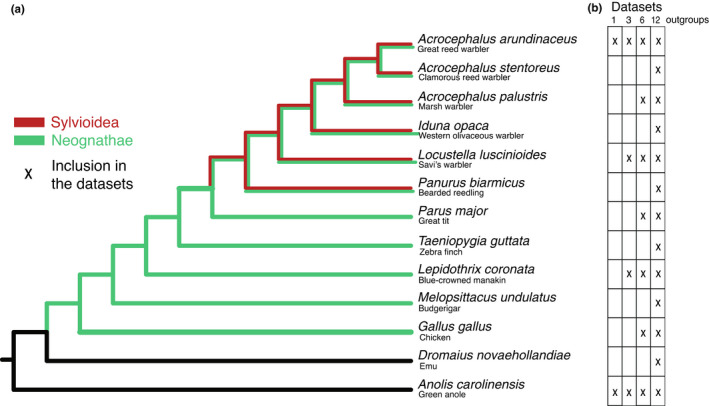
(a) Reference species topology used in the ELW and BEAST analyses. (b) Species included in the analyses of how lowering the number of species affected the *t*_RC_ estimates in the BEAST analysis.

We evaluate the ELW and BEAST approaches along with the synonymous substitution rate (dS) approach, and a maximum likelihood collapsed tree (ML_CT_) approach, using sequence data from several Z and W gametologs of the great reed warbler (*Acrocephalus arundinaceus*) and related species in the Sylvioidea superfamily as well as several outgroups (Figure 1). We focus on the Sylvioidea clade because they have a pair of neo‐sex chromosomes that consists of an ancestral part that has been sex‐linked since the origin of birds ca. 150 Myrs ago, and a previously autosomal part that was added to the sex chromosomes by a fusion event ca. 24 Myrs ago (Sigeman et al., 2020, 2021). This provides us with testable predictions to which the different approaches to estimate *t*_RC_ can be evaluated: *t*_RC_ for genes on the added part should not predate the origin of the Sylvioidea clade, and *t*_RC_ of genes on the ancestral part should consistently be older than for genes on the added part. We conclude that the ELW and BEAST approaches perform similarly and that both outperform the dS and ML_CT_ approaches. Additionally, we show that the precision of estimating *t*_RC_ with ELW can be improved by including W gametologs from more species and evaluate how decreasing the number of outgroups affects the BEAST approach. The scripts providing commands and options used for the programmes are available on a GitHub repository (https://github.com/HKyleZhang/tRC_estimation.git).

## 2 METHODS

### 2.1 Dataset and reference species topology

#### 2.1.1 Dataset generation

Reads from a previously described Illumina paired‐end dataset (accessible on NCBI under BioProject ID PRJNA578893) of one individual of each sex of six Sylvioidea species—great reed warbler (*Acrocephalus arundinaceus*), clamorous reed warbler (*A. stentoreus*), marsh warbler (*A. palustris*), western olivaceous warbler (*Iduna opaca*), Savi's warbler (*Locustella luscinioides*), and bearded reedling (*Panurus biarmicus*)—were aligned to a high‐quality *A. arundinaceus* reference genome (Sigeman et al., 2021) in which W‐linked scaffolds were excluded. The W scaffolds were excluded because W chromosomes diverge and degrade rapidly, which means that reads of other species may align poorly to them (a problem that increases with increasing time since speciation and recombination cessation). For each of these six species, we extracted the Z and W exonic sequences of 147 genes based on female‐specific (i.e., W‐specific) SNP distributions. In a nutshell, we called W variants when the male was homozygous and the female heterozygous (for more details on methodology including filtering criteria for trimming reads and calling variants, see Sigeman et al., 2019, 2021). Our previous work confirms that extracting female‐specific (W‐specific) SNPs based on one individual of each sex provides almost identical results compared with using two individuals of each sex, owing to usually many more W‐specific alleles than there are polymorphic sites on the Z (Sigeman et al., 2019, 2021; H. Sigeman and B. Hansson unpublished). All exonic sequences were aligned with PRANK v.140603 (options: ‐f = fasta ‐F; Löytynoja, 2014) and concatenated using the program catfasta2phyml (https://github.com/nylander/catfasta2phyml/; last accessed September 24, 2021).

Next, we added one‐to‐one orthologues (downloaded from the Ensembl BioMart database; Smedley et al., 2015) from seven outgroup species: great tit (*Parus major*), zebra finch (*Taeniopygia guttata*), blue‐crowned manakin (*Lepidothrix coronata*), budgerigar (*Melopsittacus undulatus*), chicken (*Gallus gallus*), emu (*Dromaius novaehollandiae*), and green anole (*Anolis carolinensis*). Notably, these orthologues were either Z‐linked or autosomal as the added part of the neo‐sex chromosome in Sylvioidea corresponds to an autosome in non‐Sylvioidea species. Also, we did not include W gametologs from the outgroup species. We aligned these orthologues to the Sylvioidea gene alignments using MAFFT with options: –add –reorder –auto. Then, we removed ambiguous sites ‘N’ and trimmed gaps with Gblocks v.0.91b (options: ‐t = c ‐p = y ‐e = .gb; Castresana, 2000) in the alignments. After retaining alignments where all 13 species were present and the alignment length was >500 bp, we manually examined each alignment and removed three genes on the ancestral part, as these genes showed high similarities between Z and W sequences, suggesting the true W sequences were not extracted (potentially they are lost). Eventually, we obtained a dataset of 51 genes, including 22 genes on ancestral part and 29 genes on added part of the neo‐sex chromosome.

#### 2.1.2 Reference species topology

We gathered the phylogenetic relationships between the 13 species to reconstruct a reference species topology (Figure 1a; Alström et al., 2013; Jarvis et al., 2014; Oliveros et al., 2019). Of the 13 species, all except one Paleognath (*D. novaehollandiae*) and one reptile (*A. carolinensis*) are Neognaths (six Sylvioidea species: *A. arundinaceus*, *A. stentoreus*, *A. palustris*, *I. opaca*, *L. luscinioides*, and *P. biarmicus*, and five non‐Sylvioidea species: *P. major*, *T. guttata*, *L. coronate*, *M. undulatus*, and *G. gallus*).

### 2.2 Estimating *t*_RC_ using the dS, ML_CT_ , ELW, and BEAST approaches

#### 2.2.1 Synonymous substitution rate (dS) approach

For each gene alignment, the synonymous substitution rate (dS) was estimated using Codeml (Yang, 2007) with a one ratio model. We used an in‐house R script to obtain the dS between the Z and the W gametologs of *A. arundinaceus* from the maximum likelihood pairwise comparisons in the output.

#### 2.2.2 Maximum likelihood collapsed tree (ML_CT_ ) approach

For each gene alignment including sequences of the 13 species and the W gametolog of *A. arundinaceus*, ML trees were reconstructed using RAxML version 8.2.12. (Stamatakis, 2014) with options: ‐m GTRGAMMAX ‐f a ‐p 20210504 ‐x 20210504 ‐N autoMRE. We collapsed ML tree branches with bootstrap value <0.7, and the collapsed trees were drawn in cladograms.

The collapsed trees were compared with the reference species topology indexed on the biologically meaningful phylogenetic positions for recombination cessation. The tree position range where the *A. arundinaceus* W gametolog branched off from the collapsed tree was assigned manually.

#### 2.2.3 Expected likelihood weight (ELW) approach

**FIGURE 2:**
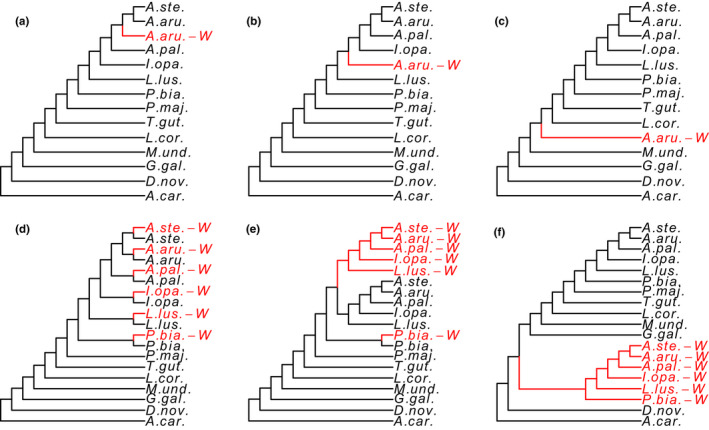
Examples of hypothetical topologies of Z and W gametologs under different recombination cessation scenarios when only including the *A. arundinaceus* W gametolog (a–c; the single‐W dataset) or when including W gametologs of all six Sylvioidea species (d–f; the multi‐W dataset). (a) Hypothetical topology no. 2 indicating recombination cessation before *A. arundinaceus* and *A. stentoreus* diverged, but after the split of these two species and *A. palustris*; (b) hypothetical topology no. 4 indicating recombination cessation before the speciation of *I. opaca* and after *L. luscinioides*; (c) hypothetical topology no. 9 indicating recombination cessation earlier than the formation of Sylvioidea, before the speciation of *L. coronata* and after *M. undulatus*; (d) hypothetical topology no. 1 indicating continuation of recombination after speciation of each Sylvioidea species; (e) hypothetical topology no. 5 indicating recombination cessation before speciation of *L. luscinioides* and after *P. biarmicus*; (f) hypothetical topology no. 11 indicating recombination cessation before *G. gallus* and after *D. novaehollandiae* speciation.

In gene trees based on sequences including both Z and W gametologs, recombination cessation at different time points will generate different topologies (Figures 2, S1, S2). We constructed 12 hypothetical topologies representing different recombination cessation scenarios for alignments including only the W gametolog of *A. arundinaceus* (single‐W dataset; Figures 2a–c, S1), and for alignments including the W gametologs of *A. arundinaceus* and 5 additional Sylvioidea species (multi‐W dataset; Figures 2d–f, S2). The 12 hypothetical topologies were ordered and indexed from recent (no. 1) to ancient recombination cessation (no. 12). We did not include the scenarios where the W gametologs diverged before the bird–reptile split.

For each gene alignment, we computed ELW values for the 12 hypothetical topologies by using the topology testing analysis in IQ‐Tree version 2.1.2 (Minh et al., 2020) with the following options: ‐s [alignment] ‐‐trees [hypothetical topologies set] ‐‐test 100000 ‐‐test‐au. This resulted in an array of 12 ELW values representing the distribution of likelihood weights across different *t*_RC_. We did these analyses separately for the single‐W and the multi‐W datasets using the same settings.

#### 2.2.4 BEAST approach

For each gene alignment including sequences of the 12 outgroup species and the Z and W gametologues of *A. arundinaceus*, BEAUTi (Bouckaert et al., 2019) was used to configure a TN93 + G4 + I site model with relaxed log‐normal clock (Drummond et al., 2006), and specified calibration priors for all the nodes except for the divergence node between Z and W gametologues of *A. arundinaceus* (Table S1). BEAST2 version 2.6.3 (Bouckaert et al., 2019) was used to run MCMC with a chain length of 200 000 000 and a pre‐burnin of 10 000 steps. Samples were taken every 10 000th step. We used the *tracerer* R package (Bilderbeek & Etienne, 2018) to evaluate the effective sample size and obtain the posterior median, and 95% highest posterior density (HPD), of the posterior probability distribution for the divergence node between Z and W of *A. arundinaceus*.

To evaluate how lowering the number of outgroups affected the precision of *t*_RC_, we generated three additional datasets that included, in addition to the *A. arundinaceus* Z and W gametologs, sequences of 6, 3, and only 1 outgroup species (Figure 1b). The configuration in BEAUTi was adjusted according to the number of outgroups, but other settings were retained. The results from these additional datasets were then compared with each other and to the results from the original dataset that included 12 outgroups.

### 2.3 Statistics

To evaluate these four approaches for estimating *t*_RC_, we employed a clustering analysis assuming that the genes on the ancestral and added part of the neo‐sex chromosome formed two clusters a priori. For the *t*_RC_ estimates of the dS, ML_CT,_ and ELW approaches, K‐means clustering was conducted on the Euclidean distance matrix between genes. For the *t*_RC_ estimates of the BEAST approach, K‐means clustering was conducted on the matrix of second‐order Wasserstein distance between genes, because Euclidean distance is not applicable for probability distributions. The statistical analyses were done in R. Specifically, Euclidean distance calculation and K‐means clustering were done with *stats* R package (R Core Team, 2020); Wasserstein distance calculation was done with *transport* R package (Schuhmacher et al., 2019); and handling and reformatting of the input data tables were done with *tidyverse* R package (Wickham et al., 2019).

Spearman's rank correlations were performed to test correlations between the output of different analyses and datasets; (i) between the *t*_RC_ estimates corresponding to the highest ELW value in the ELW analysis and the posterior median in the BEAST analysis, and (ii) pairwise between the posterior medians in BEAST analyses with different numbers of outgroups. The Spearman's rank correlation in (i) was done with the *stats* R package and results were visualized with *ggplot2* in the *tidyverse* R package (Wickham et al., 2019). The pairwise Spearman's rank correlations in (ii) and results visualization were done with the *GGally* R package (Schloerke et al., 2021).

Kruskal–Wallis tests and pairwise Wilcoxon signed‐rank tests were performed to compare the width of 95% HPD interval of the *t*_RC_ estimates of BEAST analyses with different numbers of outgroups. Kruskal–Wallis tests and Wilcoxon signed‐rank tests were done with the *stats* R package and results were visualized with *ggplot2* in the *tidyverse* R package (Wickham et al., 2019). Bonferroni corrections were performed when there were multiple tests.

## 3 RESULTS

### 3.1 Estimating *t*_RC_ using the dS, ML_CT_ , ELW, and BEAST approaches

The dS, ML_CT_, ELW, and BEAST approaches were evaluated with the data of 22 genes on the ancestral part, and 29 genes on the added part, of the Sylvioidea neo‐sex chromosome. Considering that the added part fused ca. 125 Myrs after the formation of the ancestral sex chromosome, we assumed two spatial clusters of genes differing in *t*_RC_.

**FIGURE 3:**
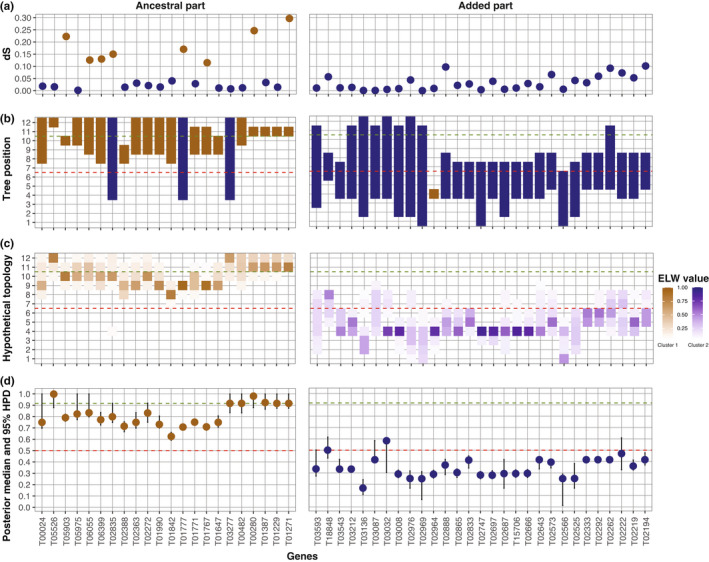
Estimates of *t*_RC_ across 51 neo‐sex chromosome genes from (a) the dS approach, (b) the ML_CT_ approach, (c) the ELW approach, and (d) the BEAST approach. Genes were ordered according to the physical positions on *A. arundinaceus* neo‐Z, and included 22 genes from the ancestral part and 29 genes on the added part (Sigeman et al., 2021). Colors correspond to clusters of a K‐means clustering analysis (*k* = 2), with brown indicating Cluster 1 and blue Cluster 2. The color gradient (heatmap) in the ELW approach indicates the ELW values for each hypothetical topology. The two horizontal lines mark the origin of Neognathae (green) and Sylvioidea (red).

For the dS approach, which was evaluated using Z and W gametologs of *A. arundinaceus*, we found dS values between 0.0001 and 0.2973. However, there was a considerable overlap in dS values between genes located on the ancestral and added parts of neo‐sex chromosome. The clustering analysis showed that eight genes of the ancestral part (T05903, T06055, T6399, T02835, T01777, T01767, T00280, and T01271) formed Cluster 1, whereas the other 14 genes of the ancestral part were grouped with all of the 29 genes of the added part in Cluster 2 (Figure 3a).

For the ML_CT_ approach, based on sequences of the 12 species in addition to the Z and W gametologs of *A. arundinaceus*, many genes showed imprecise tree positions. The clustering analysis grouped 19 genes of the ancestral part and 1 gene of the added part in Cluster 1, whereas the other three genes of the ancestral part (T02835, T01777, and T03277) and 28 genes of the added part were grouped in Cluster 2 (Figure 3b). Three genes of the ancestral part (T02835, T01777, and T03277) showed a tree position range between 4 and 12 (Figure 3b), implying that continued recombination after the formation of Sylvioidea could not be excluded. On the added part, no fewer than 27 genes had a tree position range exceeding 6.5 (Figure 3b), indicating the erroneous possibility of recombination cessation even before the chromosome fusion took place.

In contrast to the dS and ML_CT_ approaches, the ELW and BEAST approaches separated clearly between genes of the ancestral and added part of the neo‐sex chromosome by grouping them in Cluster 1 and 2, respectively (Figure 3c,d). However, both approaches gave some uncertainty in the *t*_RC_ estimates (Figure 3c,d). With the ELW approach, the range of likely topologies overlapped the origin of the Sylvioidea clade for 1 gene on the ancestral part (T02835; Figure 3c) and for 16 genes on the added part. The BEAST approach showed fewer uncertainties, with only four genes on the added part having a 95% HPD interval spanning the origin of Sylvioidea (Figure 3d, S3).

**FIGURE 4:**
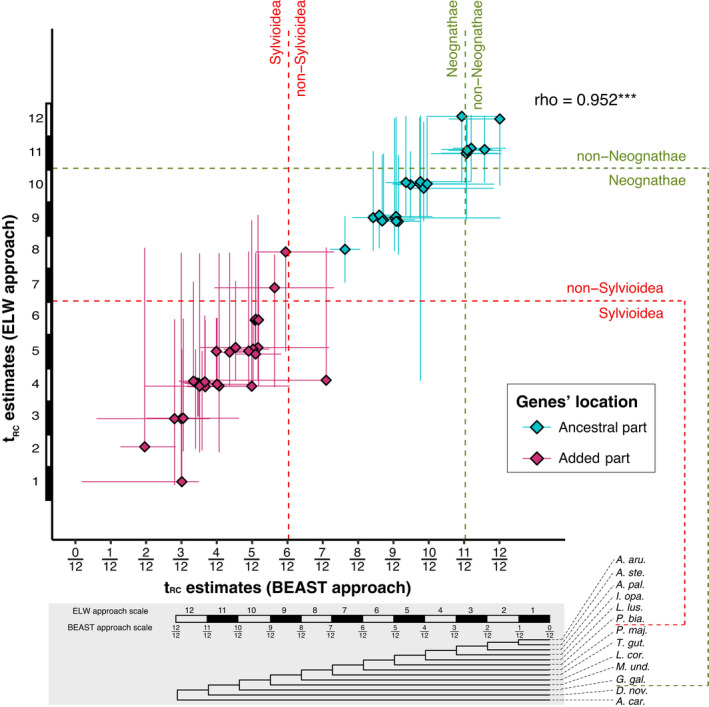
Relationship between estimates of *t*_RC_ of the BEAST and ELW approaches plotted on a convertible scale corresponding to the posterior median and 95% HPD for the BEAST approach and the highest ELW‐value topology and topology range of 95% accumulated ELW values for the ELW approach. Genes are colored according to their physical position on the ancestral (turquoise) or added (pink) part of the neo‐sex chromosome of *A. arundinaceus*. The *t*_RC_ estimates of the ELW and BEAST approaches were significantly correlated (Spearman's rank test: *ρ* = 0.952; ***: *p* < 0.001). The scale gives the conversion of the *t*_RC_ estimates and the corresponding phylogenetic positions on the reference species topology. The timings for the origin of Neognathae (green) and Sylvioidea (red) are marked with dashed lines.

The most probable *t*_RC_ estimates in the ELW and BEAST analyses were highly correlated (Spearman's rank correlation: *ρ* = 0.952, *p* < 0.001; Figure 4). Considering the most likely *t*_RC_ estimates of the two approaches (i.e., the hypothetical topology with the highest ELW value, and the posterior median, respectively), we found that both approaches identified the same few genes with recent recombination cessation on the added part (left lower corner in Figure 4), and the same few genes with ancient recombination cessation on the ancestral part (right upper corner in Figure 4). However, the ELW approach identified the most likely *t*_RC_ estimate of two genes, and the BEAST approach of one gene, on the added part to be earlier than the fusion event per se indicated by their *t*_RC_ in relation to the origin of Sylvioidea (Figure 4).

### 3.2 Estimating *t*_RC_ with the multi‐W dataset using the ELW approach

**FIGURE 5:**
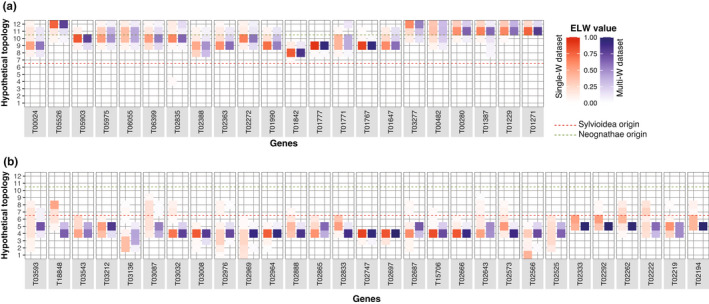
ELW values for each hypothetical topology when using the single‐W dataset (red heatmap) and the multi‐W dataset (blue heatmap), respectively, for genes on (a) the ancestral part and (b) the added part of the neo‐sex chromosome.

The ELW approach was further applied to compare the estimation of *t*_RC_ using the single‐W dataset (including only the W gametolog of *A. arundinaceus*) and the multi‐W dataset (including W gametologs of six Sylvioidea spp.; see Figure 2d–f). The genes of the ancestral part of the neo‐sex chromosome were only affected marginally by including more W gametologs; the estimated *t*_RC_ (i.e., the hypothetical topology with the highest ELW value) and the range of *t*_RC_ (i.e., the range of hypothetical topologies) differed only slightly for these genes between these two datasets (Figure 5a). In contrast, for several genes of the added part, the multi‐W dataset yielded a narrower *t*_RC_ range and a higher ELW value for the most likely hypothetical topology. Notably, all of the genes on the added part had lowered uncertainties of *t*_RC_ indicating recombination cessation before the origin of Sylvioidea with the multi‐W dataset (Figure 5b).

### 3.3 Estimating *t*_RC_ with different numbers of outgroups in the BEAST approach

**FIGURE 6:**
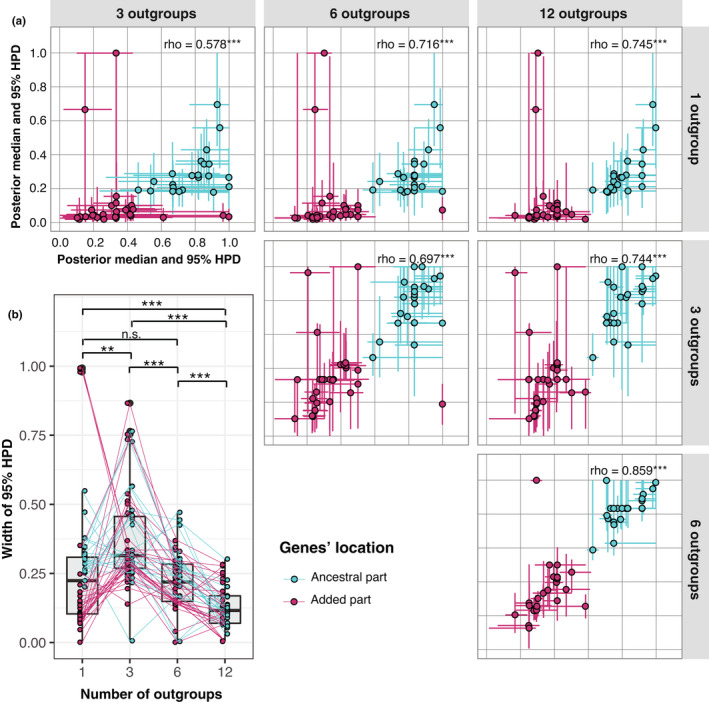
(a) Posterior medians and 95% HPDs, and (b) the width of 95% HPD, for BEAST analyses of the 1‐, 3‐, 6‐, and 12‐outgroup datasets, respectively. Colors indicate the genes' location on the ancestral (turquoise) or added (pink) part of the neo‐sex chromosome. In (a), Spearman's rank correlation coefficients and their significance levels are given (***: *p* < 0.001). In (b), overlaid on the box plots (grey), each gene is connected with lines between datasets, and significant values from post hoc analyses with Wilcoxon signed‐rank tests after correction for multiple comparisons are given as followed: n.s.: not significant; **: *p* < 0.01; ***: *p* < 0.001.

We evaluated the performance of the BEAST approach when lowering the number of outgroups from the original 12 species to 6, 3, and 1, respectively (Figure 1b). Overall, the posterior medians were significantly correlated after lowering the number of outgroups (Spearman's rank correlations: *p* < 0.001 in all six pairwise comparisons after correction for multiple comparisons), and even the analysis with 1 outgroup separated most genes on the ancestral and added parts of the neo‐sex chromosome (Figure 6a). However, the 1‐outgroup dataset resulted in systematic underestimation of *t*_RC_ compared with the other datasets (Figure 6a). The width of 95% HPD, which indicates the uncertainty of the *t*_RC_ estimates, had an overall significant difference between datasets (Kruskal–Wallis tests: *p* < 0.001). The median of the 95% HPD width was largest for the 3‐outgroup dataset, and smallest for the 12‐outgroup dataset, and a post hoc analysis showed significant differences between all pairwise comparisons (Wilcoxon signed‐rank tests: *p* < 0.01–0.001) except for the comparison between the 1‐ and 6‐outgroup datasets (*p* = 1.000; Figure 6b).

## 4 DISCUSSION

Recombination cessation plays a crucial role for the evolutionary transition of old and often highly differentiated sex chromosomes from their ancestral autosomal stage (Bachtrog et al., 2011; Charlesworth et al., 2005; Wright et al., 2016). Studying how recombination cessation proceeds over the sex chromosomes, thereby increasing our insight into sex chromosome evolution, requires reliable estimates of *t*_RC_. Estimating *t*_RC_ is frequently done using dS or ML trees, but these approaches may come out with imprecise, or even incorrect, inferences of *t*_RC_ for several different reasons. For example, the dS approach assumes neutrality of all synonymous substitutions and molecular clock‐like evolution of the gametologs, which is not necessarily achieved due to codon usage bias, GC content variation, gene conversion bias and site‐specific mutation rates. Therefore, we introduce and assess two alternative phylogenetic approaches, the ELW and BEAST approaches, with the aim to pinpoint the most probable *t*_RC_, and to improve the assessment of *t*_RC_ by transforming the uncertainty of the topology of a gene tree to an uncertainty around *t*_RC_. To our knowledge, the performance of different approaches to estimate *t*_RC_ has not yet been evaluated, which makes it difficult to interpret the results from analyses of *t*_RC_ and eventually demarcate evolutionary strata.

In this study, we took advantage of the Sylvioidea neo‐sex chromosome as the contrasting age between the ca. 150 Myr‐old ancestral part and the ca. 24 Myr‐old added part provides us with testable predictions to evaluate the different approaches to estimate *t*_RC_. To achieve an unbiased assessment of all four approaches, we used a clustering analysis to scrutinize which approaches were able to give *t*_RC_ estimates by which the genes from the two chromosome regions were grouped into separate clusters. We found that the *t*_RC_ estimates from the ELW and BEAST analyses managed to group the genes from the different regions in separate clusters, on the contrary to the dS and ML_CT_ analyses. With the dS approach, genes with relatively high dS values (*n* = 8 genes), occurring exclusively in the ancestral part, formed the first cluster, whereas genes with relatively low dS from both regions were grouped into the second cluster (*n* = 43 genes, including 14 genes on ancestral part and 29 genes on added part). With phylogenetic estimation of *t*_RC_, such as with the ML_CT_ approach we have applied here, the confidence of the estimates depends on the statistical support of the relevant branches in the gene tree. We collapsed branches with low bootstrap support (<0.7), thereby allowing polytomies. As shown by the clustering analysis, the ML_CT_ approach improved the extent to distinguish the ancestral and added parts in terms of *t*_RC_ compared with the dS approach, but four genes were wrongly classified (Figure 3b). Three of the four wrongly classified genes were located on the ancestral part and had wide tree position ranges (T02835, T01777, and T03277) spanning tree positions from 4 to 12, similar to many genes on the added part that also had wide ranges. The other wrongly classified gene was located on the added part (T02964) and showed a narrow tree position range, that is, 4, which in turn accounted for its unexpected clustering with the majority of genes on the ancestral part of which some also have narrow tree position ranges (Figure 3b). We believe that this is caused by the fact that we clustered genes by using Euclidean distances computed as single values from multi‐dimensional vectors. Such scaling of a high dimensional space is always likely to lead to lost information to some extents, in this case giving similar distance‐values to some genes with narrow tree position range despite differing in tree position.

Compared with collapsing branches to obtain a supported but often blunt topology as in the ML_CT_ approach, the ELW and BEAST approaches utilize a reference species topology and provide the most likely and a range of possible *t*_RC_. The ELW approach analyses the specific set of hypothetical topologies that together represent all possible recombination cessation time points. The topology with the highest ELW value gives the most likely topology and *t*_RC_, and the ELW values of the other topologies provide a straightforward indication of the uncertainty for the most likely *t*_RC_ (Figure 3c). Similarly in the BEAST approach, the posterior median gives the most probable *t*_RC_ and the 95% HPD indicates the uncertainty (Figure 3d).

Both the ELW and the BEAST approach were able to distinguish the genes of the ancestral and added parts as separate clusters (Figure 3c,d), and their *t*_RC_ estimates were well correlated (Figure 4). Nevertheless, they resulted in varying degrees of uncertainty of the *t*_RC_, with the ELW approach having more genes with larger degree of uncertainty. This may be explained by the different set‐ups between two approaches. For the ELW approach, the estimation is based on a finite set of hypothetical topologies, so that possible *t*_RC_ estimates are finite and discrete, and dependent on the number of species. In contrast, the BEAST approach provides continuous *t*_RC_ estimates between 0 and 1, and would thus in theory have better resolution. Considering the convertible scales between two approaches, the discrete and finite *t*_RC_ estimates of the ELW approach are separated by 13 values of *t*_RC_ estimates in the BEAST approach (Figure 4). Besides the overall coherence between the ELW and BEAST approaches, we spotted some incongruences. For example, the T03032 gene on the added part had the highest ELW value on hypothetical topology no. 4 indicating that recombination most likely ceased after the speciation of *L. luscinioides* and before the speciation of *I. opaca*. In contrast, with the BEAST approach, this gene had a posterior median larger than 6/12, suggesting the unlikely recombination cessation event earlier than the origin of Sylvioidea. However, the reported uncertainties for this gene by both approaches overlapped and included possible *t*_RC_ after the origin of Sylvioidea. In general, erroneous calling of W variants will affect the *t*_RC_ estimates. For example, interpreting Z polymorphisms as W‐specific sites will overestimate *t*_RC_ to be more ancient than it should be. We called W variants based on a single individual of each sex, but we believe that this did not inflate our *t*_RC_ estimates as we have found almost identical W variants when using two individuals of each sex (Sigeman et al., 2019, 2021; H. Sigeman and B. Hansson unpublished).

Phylogenetic approaches can be improved by increasing the number of sequences in the alignment, such as including W gametologs of more species or sequences of more species in the reference species topology. We found that the ELW analyses based on the single‐W dataset can overestimate *t*_RC_, as often seen in the ML_CT_ approach, thereby erroneously indicating that the added part of neo‐sex chromosomes should have ceased to recombine before the origin of Sylvioidea. This could have been caused by long‐branch attraction or some similar process. Long‐branch attraction is a systematic error in phylogenetic inference that arises when there is fast evolution in some lineages which leads to many unique substitutions and similarities between sequences that are not related by descent (Bergsten, 2005). Genes on Y and W chromosomes are affected by both genetic drift and purifying selection, and, due to strong linkage, genetic hitchhiking (Bachtrog, 2013). How these processes affect specific genes vary, for example, in relation to their degree of essential functionality, dose dependence, and chromosomal location, but it is clear that many W genes evolve differently compared to autosomal and Z‐linked genes (Mank et al., 2010; Sigeman et al., 2021). It is possible that the W sequences of *A. arundinaceus* have accumulated substitutions faster and somewhat differently than the other non‐W sequences in the alignments, which in turn predisposed them to long‐branch attraction‐like errors. It seems that this problem was largely controlled when W sequences from multiple species were included in the ELW analysis, which rendered more correct and precise *t*_RC_ estimates.

As the resolution in the estimates of *t*_RC_ with the ELW approach depends on the time between speciation events, it is important to carefully select species with relevant time intervals for the reference species topology. Increasing the number of species provides a larger set of hypothetical topologies to be evaluated and shortens the time intervals between the speciation events. This may increase the resolution of the analyses, but only if the ELW values in this larger set of hypothetical topologies are sufficiently distinct. We evaluated how phylogenetic estimation of *t*_RC_ with the BEAST approach can be affected by lowering the number of outgroups from the 12 to 6, 3, and only 1. This showed that the posterior median was not much affected until the number of outgroups dropped to 1, for which most genes had unreasonably low values (Figure 6a). However, a few genes showed deviating posterior medians in the 3‐ and 6‐outgroup analyses (Figure 6a), and in general the uncertainty of the *t*_RC_ estimates as measured by the width of the 95% HPD decreased when more outgroups were included (Figure 6b). Unexpectedly, the median of the width of the 95% HPD peaked when 3 outgroups were included, whereas some extremely narrow 95% HPD widths in 1‐outgroup analysis probably reflect the low, near‐zero, posterior medians at many genes.

Previous studies have shown that the ancestral part of the neo‐sex chromosomes of Sylvioidea birds ceased to recombine before the origin of Sylvioidea except for the small pseudo‐autosomal region (PAR) in the beginning of the chromosome, and that the added part has ceased to recombine over its entire length in *A. arundinaceus* and other Sylvioidea species (Pala et al., 2012; Sigeman et al., 2020, 2021). Albeit research on other avian clades suggest two to three (Paleognaths) or four strata (Passerines) on the ancestral sex chromosomes (Zhou et al., 2014; Xu et al., 2019), our results provide little support for as many as four evolutionary strata on the ancestral part of the neo‐sex chromosome in Sylvioidea passerines. However, although our aim was not to define strata at the ancestral part, we observed that genes with old recombination cessation (i.e., large *t*_RC_ estimates) occur near the distal end of the ancestral sex chromosome, which coincides with S0, the oldest stratum, in other bird species (Zhou et al., 2014; Xu et al., 2019). Notably, this pattern is more obvious for the *t*_RC_ estimates with the ELW and BEAST approaches, and to some extent the ML_CT_ approach, than with the dS approach (Figure 3).

For the added region, the present study shows that neither the ELW nor the BEAST approach could resolve the history of recombination cessation with strong confidence, based on the single‐W dataset. Specifically, *t*_RC_ estimates of most genes suggest several possible recombination cessation scenarios, which makes it difficult to affirmatively demarcate strata. When compared to the ancestral part, the added part with its significantly recent *t*_RC_ could be considered a single stratum (Figure 3c,d). However, within the added part, the *t*_RC_ estimates show a local and/or mosaic recombination suppression landscape (Figure 3c,d). Interestingly, adding W gametologs from another Sylvioidea species (the multi‐W dataset) to the ELW analysis made it possible to narrow the range of *t*_RC_ for almost all genes on the added part, compared with the analysis using a single‐W sequence (Figure 5b). Based on the more precise *t*_RC_ estimates from the multi‐W dataset, we note for the added part that (i) several genes proximate to the fusion point and in the distal end of the chromosome have experienced recombination cessation after the speciation of *P. biarmicus* and before the speciation of *L. luscinioides* (21–17 Myrs ago), and that (ii) several genes in the central region show recombination cessation after the speciation of *L. luscinioides* but before the speciation of *I. opaca* (17–7 Myrs ago). This pattern implies a complex (non‐linear) spread of recombination suppression from the ancestral to the added part.

An important result from the ELW analysis using the multi‐W dataset is that the added part did not stop recombining at or right after the fusion event (ca. 24 Myrs ago), but continued to recombine for at least 3 Myrs (recombination cessation initiated 21–17 Myrs ago). Thus, the added region could have been acting as a second PAR after becoming physically linked to the ancestral part. In other birds, a single PAR is located in the beginning of the sex chromosome (Zhou et al., 2014). Theory suggests that linkage in the form of cosegregation between sexually antagonistic alleles at different loci is a main driver of sex chromosome evolution (the sexual antagonism hypothesis; Ponnikas et al., 2018). When recombination between Z and W chromosomes continues (as in a PAR), the alleles with potential sex‐specific benefits will not segregate according to the sex, and then the potential sex‐specific beneficial associations will disappear. However, the recombination rate in the added part could have been significantly reduced after the fusion, and consequently, the segregation partially sex‐linked at least in the PAR boundary (i.e., around the fusion point). This suggestion has some circumstantial support from recent result of the recombination pattern on the Z chromosome in male *A. arundinaceus*, showing an extreme bias toward recombination in the end of the sex chromosomes, and that a large central section covering at least 78% of the total neo‐sex chromosome, including a large region of the added part, lacks recombination also in males (Ponnikas et al., 2022). Thus, it is possible that physical linkage between the ancestral sex chromosome and the previously autosomal added part caused a significant reduction in recombination around the fusion point, which may have been sufficient for driving the fusion to fixation in the ancestor of Sylvioidea in accordance with the sexual antagonism hypothesis. Alternative hypotheses for the latter include genetic drift and beneficial cosegregation between genes on each side of the fusion point for reasons unrelated to sexual antagonism and sex linkage.

In conclusion, evolutionary strata were recently challenged by data suggesting a more local and mosaic expansion of non‐recombining regions (Natri et al., 2013; Sigeman et al., 2021). The demarcation of evolutionary strata has usually been done with the dS and ML_CT_ approaches. To circumvent the potential problems with the dS approach and the difficulties in interpreting *t*_RC_ with the ML_CT_ approach, we introduced two alternative phylogenetic approaches, the ELW and BEAST approaches. The latter two approaches benefit from using a reference species topology, which makes it possible to transform the uncertainty of the topology of a gene tree to an uncertainty around *t*_RC_. We evaluated the approaches by their ability to detect a distinct difference in *t*_RC_ between the ancestral and the added part of the neo‐sex chromosome of *A. arundinaceus*. The ELW and BEAST approaches estimated *t*_RC_ with similar precision and separated clearly the *t*_RC_ of genes of the ancestral and added parts of the neo‐sex chromosome, and both outperformed the dS and ML_CT_ approaches. Our results further show that more precise *t*_RC_ estimates can be achieved by increasing the number of W sequences in the ELW analysis and including many outgroups in the BEAST analysis. Based on the *t*_RC_ estimates from the multi‐W dataset, we suggest that the added part of the Sylvioidea neo‐sex chromosome acted as a second PAR after the fusion event and has experienced a complex (non‐linear) spread of recombination suppression. We hope that our study, and the suggestion to use ELW and BEAST approaches to pinpoint the most probable *t*_RC_ for each gene, will contribute to how to define, evaluate, and understand the formation of evolutionary strata.

## AUTHOR CONTRIBUTIONS

H.Z. and B.H. conceptualized the study. H.Z. conducted the analyses with input from B.H. H.S. generated the dataset. H.Z. and B.H. wrote the manuscript with input from H.S.

## CONFLICT OF INTEREST

The authors have no conflict of interest to declare.

### PEER REVIEW

The peer review history for this article is available at https://publons.com/publon/10.1111/jeb.14068.

## Supporting information


Figure S1‐S3, Table S1

## Data Availability

The data that support the findings of this study are openly available in NCBI at https://www.ncbi.nlm.nih.gov/bioproject/?term=PRJNA578893, BioProject Accession ID PRJNA578893.
