# Supplementary material for: Assessment of phylogenetic approaches to study the timing of recombination cessation on sex chromosomes
Source: J Evol Biol. 2022 Jul 27;35(12):1721–33. doi: 10.1111/jeb.14068 (PMC10086819; doi:10.1111/jeb.14068)
Supplement: Supplementary file 1 — Figure S1‐S3, Table S1 [file JEB-35-1721-s001.docx]

# Supplementary

**
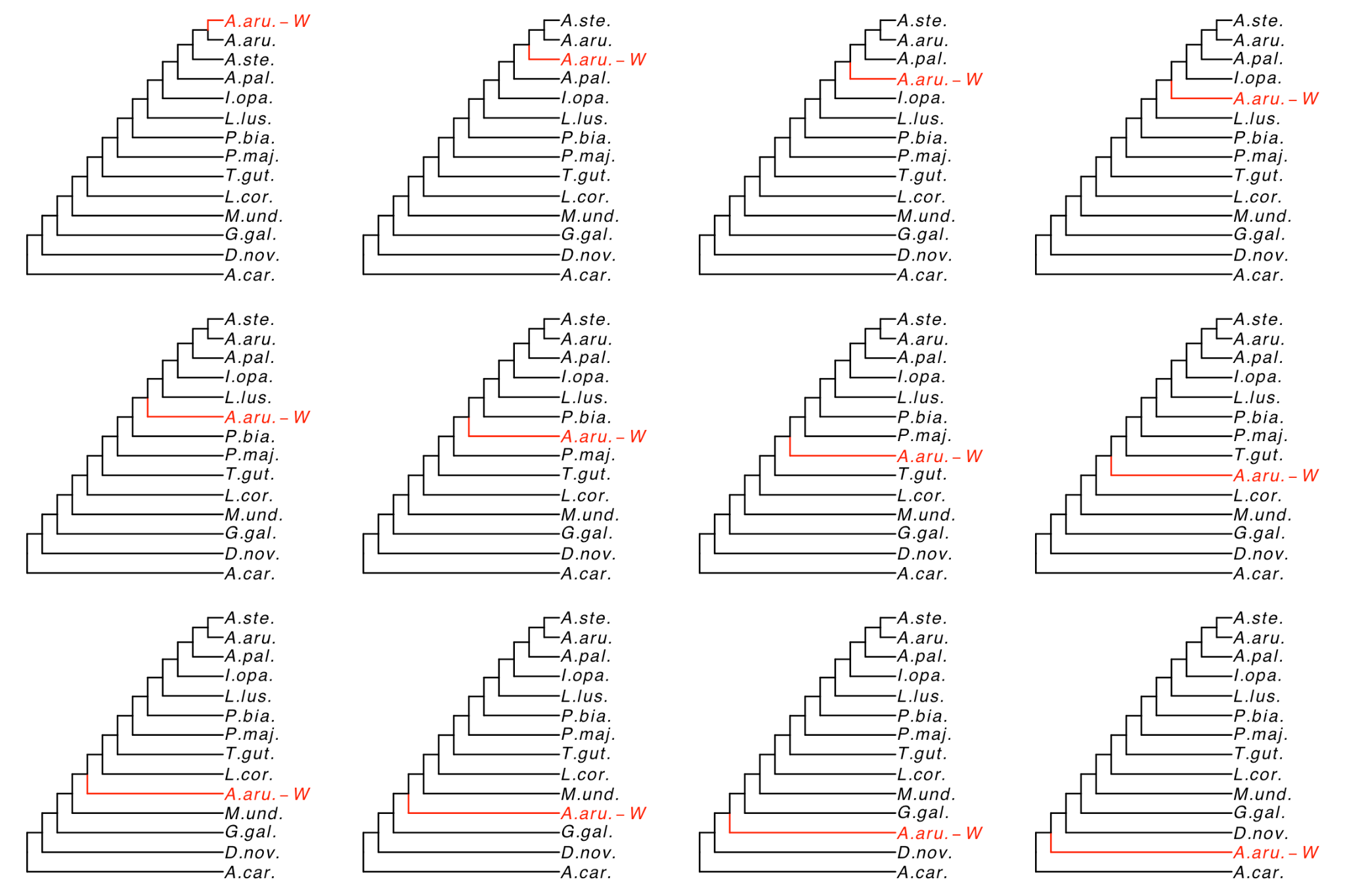
**

**Fig. S1**. Hypothetical topologies for the *A. arundinaceus*-W dataset used in the ELW analyses. The topologies range from recent (first row, left; topology no. 1) to ancient recombination cessation (third row, right; topology no. 12).


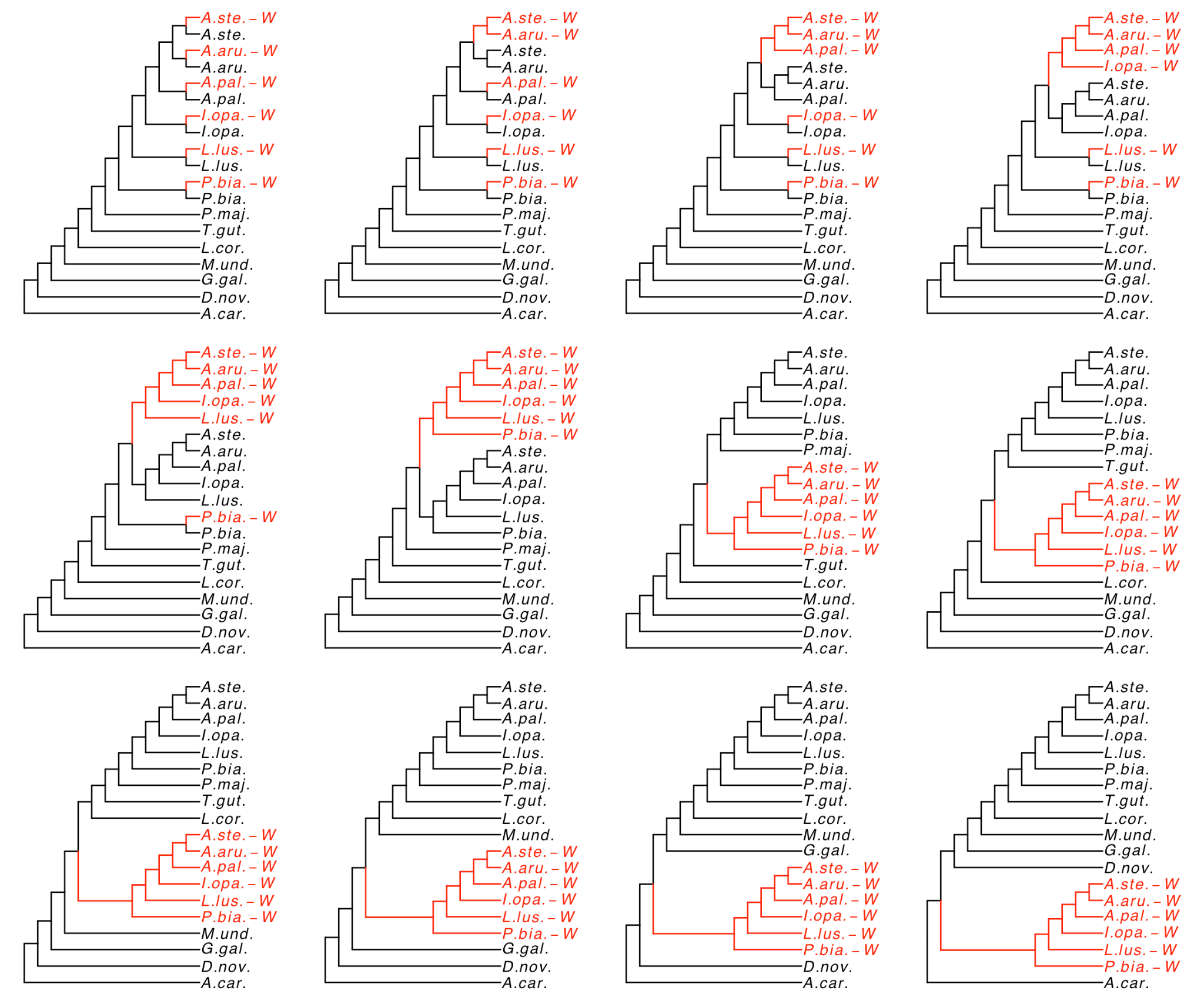


**Fig. S2**. Hypothetical topologies for the multi-W dataset used in the ELW analyses. The topologies range from recent (first row, left; topology no. 1) to ancient recombination cessation (third row, right; topology no. 12).


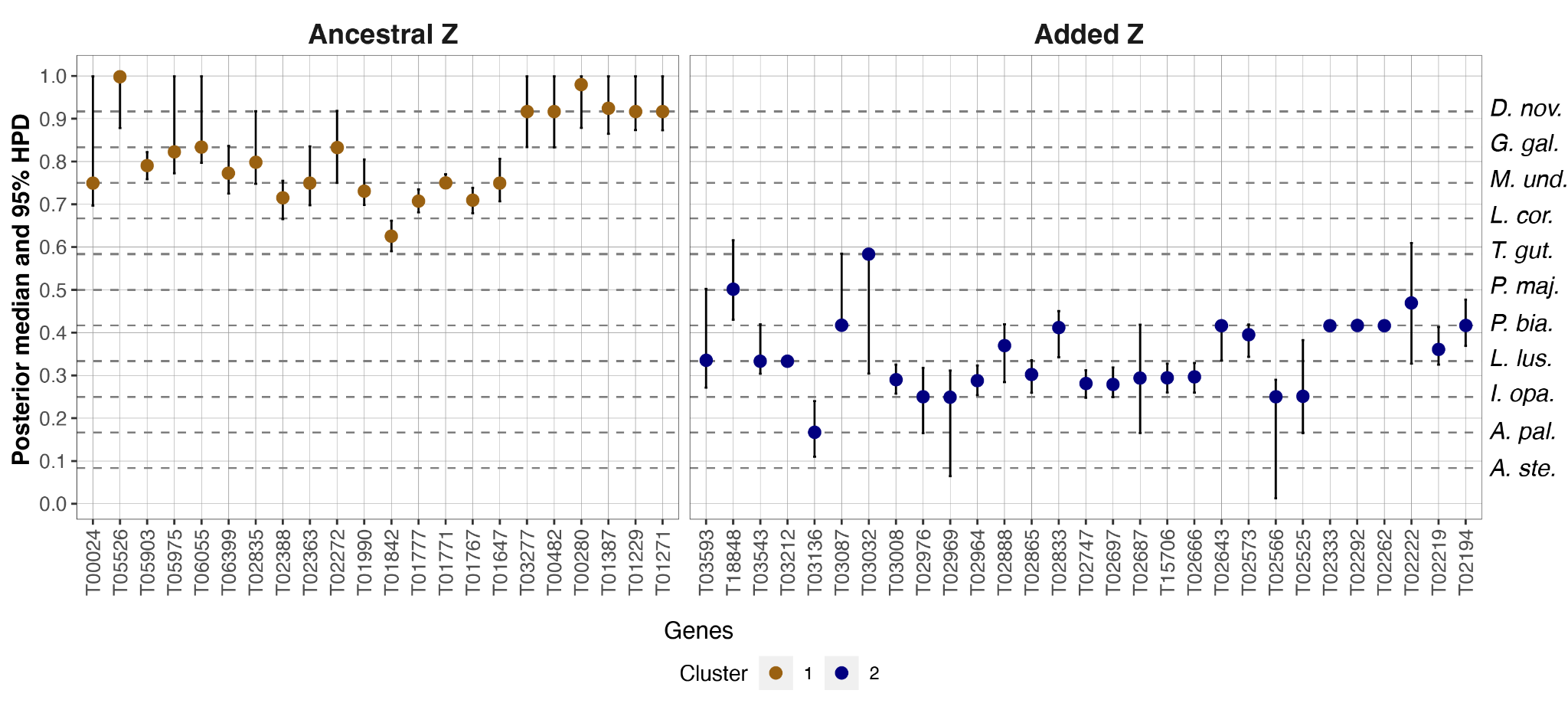


**Fig. S3**. Estimates of t_RC_ (posterior median and 95% HPD) across 51 neo-sex chromosome genes by the BEAST approach. Indicated are the speciation events for *A. stentoreus*, *A. palustris*, *I. opaca*, *L. luscinioides*, *P. biarmicus*, *P. major*, *T. guttata*, *L. coronata*, *M. undulatus*, *G. gallus* and *D. novaehollandiae*. See Fig. 3 for details.

**Table S1**. Priors used in the configuration in the BEAST analyses.

|  | | | |
| --- | --- | --- | --- |
| **Priors** | | **Detail** | **Comment** |
| Site model | | TN93 + G4 + I | All parameters in the model and nucleotide frequencies were estimated in the MCMC chain. |
| Clock model | | Relaxed log normal clock | The number of discrete rates was equal to the number of branches on the phylogenetic tree. |
| Tree | Uniform distribution | Applied on the node:  *A. aru.* \| *A. aru.*-W: U (0.001, 0.999) | Estimated in the MCMC chain for the posterior probability distribution. |
|  | Normal distribution | Applied on the nodes:  *A. aru.* \| *A. ste.*: N (1/12, 0.001)  *A. aru.* \| *A. pal.*: N (2/12, 0.001)  *A. aru.* \| *I. opa.*: N (3/12, 0.001)  *A. aru.* \| *L. lus.*: N (4/12, 0.001)  *A. aru.* \| *P. bia.*: N (5/12, 0.001)  *A. aru.* \| *P. maj.*: N (6/12, 0.001)  *A. aru.* \| *T. gut.*: N (7/12, 0.001)  *A. aru.* \| *L. cor.*: N (8/12, 0.001)  *A. aru.* \| *M. und.*: N (9/12, 0.001)  *A. aru.* \| *G. gal.*: N (10/12, 0.001)  *A. aru.* \| *D. nov.*: N (11/12, 0.001)  *A. aru.* \| *A. car.*: N (12/12, 0.001) | These nodes act as references for comparisons between genes. |
